# Lipid Peroxidation Products in Human Health and Disease 2019

**DOI:** 10.1155/2019/7147235

**Published:** 2019-11-27

**Authors:** Kota V. Ramana, Sanjay Srivastava, Sharad S. Singhal

**Affiliations:** ^1^Department of Biochemistry and Molecular Biology, University of Texas Medical Branch, Galveston, TX 77555, USA; ^2^Environmental Cardiology, University of Louisville, Louisville, KY 40202, USA; ^3^Medical Oncology and Therapeutics Research, Beckman Research Institute, City of Hope National Medical Center, Duarte, CA 91010, USA

Oxidative stress is the major cause of several life-threatening complications including various forms of cancers. Exposure of the body to external pathogens, xenobiotics, allergens, and environmental pollutants leads to increased generation of reactive oxygen species (ROS). The ROS thus generated can interact with important cellular molecules causing the disturbance in the cellular redox balance leading to various pathological consequences. One of the most important molecules directly affected by ROS is polyunsaturated fatty acids. ROS-mediated peroxidation of lipids forms various toxic lipid hydroperoxides and lipid aldehydes which act as secondary signaling intermediates in propagating the oxidative stress signals which contribute to the pathophysiology of human health and disease. Recent studies have demonstrated that the lipid peroxidation-derived lipid aldehydes regulate a number of human pathological complications including cancer, diabetes, cardiovascular, neurological, and various inflammatory diseases. Recent evidence also suggests that lipid peroxidation-derived lipid aldehydes act as biomarkers of various disease processes such as Alzheimer's and Parkinson's. Thus, continuous research work on lipid peroxidation and its generated products is very important in identifying the novel signaling mechanisms involved various human diseases and to explore possible biomarkers of disease and develop better therapeutic approaches. Through a series of special issues, we are continuously encouraging investigators to share their novel research work highlighting the significance of lipid peroxidation products in the pathophysiology of various human diseases.

In the 2019 edition of this issue, the review articles and research articles have discussed how oxidative stress and lipid peroxidation products are involved in various pathological conditions.

An excellent and informative review article by S. Soodaeva et al. described how oxidative and nitrosative stress contributes to the pathophysiology of various respiratory diseases. Specifically, the formation and significance of reactive oxygen species (ROS) and reactive nitrogen species (RNS) in the respiratory tract have been exclusively discussed and possible mechanisms through which oxidative and nitrosative stress leads to lung diseases such as asthma and COPD have been suggested. Further, they have also discussed various antioxidant strategies to control the ROS and RNS which could be beneficial in treating lung diseases.

Another review article by H. Sonowal and K. V. Ramana described the significance of lipid peroxidation-derived lipid aldehyde, 4-hydroxynonenal (HNE) in the mediation of various anti- and pro-inflammatory signaling pathways. In this article, the authors have nicely discussed the formation of HNE, its interaction with cellular biomolecules, and involvement in various inflammatory complications. They have also discussed how HNE regulates NRF2-mediated anti-inflammatory signaling leading to the expression of antioxidative defense proteins such as HO1, NQO1, and GST and also NF-*κ*B-mediated expression of proinflammatory cytokines and chemokines.

The research article by M. A. Ortega et al. investigated the role of oxidative stress and lipid peroxidation in young patients with valvular incompetence leading to chronic venous insufficiency (CVI) disease. In this cohort study involving 110 patients, the authors have analyzed plasma malondialdehyde, iNOS, eNOS, NOX1, and NOX2 in various age groups. Interestingly, they found that patients with a cutoff point of age fifty years showed increased plasma malondialdehyde, a marker for lipid peroxidation, and the expression of iNOS, NOX1, and NOX2 levels. Their results indicate that the increase in oxidative stress and lipid peroxidation reflects the characteristics of the aged CVI patient with valvular incompetence.

Another research article by A. Molfino et al. examined the circulating 19,20-epoxydocosapentaenoic acids (19,20-EDPs) levels in breast cancer patients and healthy control subjects before and after supplementation with docosahexaenoic acid (DHA) for 10 days. The serum levels of 19,20-EDPs were increased in breast cancer as well as in controls after DHA supplementation. Further, they showed that 19,20-EDP levels were lowered in breast cancer patients with BRCA1.2 mutation when compared to breast cancer patients without the mutation. The luminal A-like breast cancer patients showed increased 19,20-EDP after DHA supplementation when compared to nonluminal A breast cancer patients. These results suggest that DHA oral supplementation increases 19,20-EDPs in breast cancer patients independent of breast cancer subsets. However, BRCA1.2 mutated and luminal A-subtype breast cancer patients showed altered ability of DHA epoxidation.

A research study by T. Li et al. examined the protective role of hypoxia-inducible gene domain family member 1A (Higd1a) in high fat-induced lipotoxicity. In this study, the authors have examined the effect of oleic acid and palmitate on Higd1a expression in HepG2 and LO2 cells. Their results indicate that oleic acid and palmitate increased the expression of Higd1a in the cells and knocking down of Higd1a decreased mitochondrial transmembrane potential and caused apoptosis. Further, they found that ROS increase Higd1a expression by increasing the synergistic upregulation of HIF-1 and PGC-1a in the cells. These results suggest a novel protective role of Higd1a in protecting the cells from high fat-induced oxidative stress.

L. Micheli et al. examined the seminal levels of ghrelin, obestatin, malondialdehyde (MDA), glutathione (GSH) and oxidized glutathione (GSSG), IL-6, and TNF-alpha in infertile patients with varicocele or leukocytospemia. In this study, 32 patients with leukocytospemia, 24 with variocele, and 14 control fertile subjects were recruited to examine their semen parameters. The results indicate that when compared to controls, infertile patients with leukocytospemia and variocele showed increased sperm apoptosis, IL-6, and TNF-alpha levels and decreased ghrelin, obestatin, and GSH/GSSG ratio. Similarly, lipid peroxidation aldehyde MDA levels were increased in leukocytospemia and variocele patients when compared to controls. The significance of lipid peroxidation with the etiology of tumorigenesis was reported by Y. Zhang et al. in their research article. In this study, using mouse embryonic fibroblasts, the authors have shown that 15-hydroperoxy-eicosatetraenoic acid (15s-HpETE) causes reversible oxidation of phosphatase and tensin homolog deleted on chromosome 10 (PTEN). They have also observed oxidative dimerization of thioredoxin in the cells. Further, loss of peroxiredoxin III increased 15s-HpETC-induced PTEN oxidation.

A cross-sectional study by S. R. Carneiro et al. has demonstrated the relationship between the lipid peroxidation and physical activity in women and the squamous intraepithelial lesion (SIL) of the cervix. Two groups of 18 SIL patients and 28 control subjects were compared. The lipid peroxidation marker MDA was found higher in SIL patients when compared to controls. At the same time, they also found that the SIL group patients showed a lowered international physical activity questionnaire (IPAQ) score. This study indicates the importance of physical activity in regulation of oxidative stress in patients with SIL.

Another cross-sectional study by S.-S. Wu et al. examined the serum markers of oxidative stress in relation to carotid intima-media thickness (IMT) in patients with metabolic syndrome. In this study, 134 patients with varied metabolic syndromes were examined for their plasma oxidative stress markers. They found that MDA levels and uric acid levels were associated with carotid IMT patients with varied metabolic syndrome scores. These results suggest that determining the MDA levels along with uric acid levels may be promising while monitoring carotid IMT in patients with varied metabolic syndromes.

In conclusion, it is clear from the recent findings that oxidative stress-generated lipid peroxidation products could be potential biomarkers of a broad spectrum of diseases. Therefore, thorough understanding of lipid peroxidation processes and their secondary products is important for early detection of various pathophysiological states and their complications. This could be really important in developing potential therapeutic intervention to combat debilitating diseases such as neurological, aging, cardiovascular, and cancer, where oxidative stress is implicated in the etiology of the disease.

